# Sustainable development goals and mental health: learnings from the contribution of the FundaMentalSDG global initiative

**DOI:** 10.1017/gmh.2016.20

**Published:** 2016-09-09

**Authors:** N. Votruba, G. Thornicroft, the FundaMentalSDG Steering Group

**Affiliations:** 1 Centre for Implementation Science, Institute of Psychiatry, Psychology and Neuroscience, King's College London, London SE5 8AF, UK; 2 Centre for Global Mental Health, King's College London, Institute of Psychiatry, Psychology and Neuroscience, London SE5 8AF, UK

**Keywords:** Global mental health, implementation, policy and systems, evidence translation, low and middle income countries

## Introduction

In September 2015, mental health was included in the UN Sustainable Development Goals (SDGs). In this historic step, the United Nations (UN) acknowledged the burden of disease of mental illness, and defined mental health as a priority for global development for the next 15 years. On the road to this achievement, many individuals and organisations have played a role in contributing to the inclusion of mental health in the SDGs, one of which is the global initiative called FundaMentalSDG. This group has urged the UN to include mental health in the new development goals, targets and indicators.

## The need for a global initiative to strengthen mental health in the SDGs

For the past 15 years, from the inception of the Millennium Development Goals (MDGs) to their target year of 2015, the situation for mental health has been bleak in global development. Despite the MDG's success in reducing the overall health gap between rich and poor countries, and considerable achievements for infectious diseases such as malaria or HIV/AIDS, this last generation of development goals did not include any reference to mental illness, despite the global impact of these conditions, and their cross-cutting impact on eight of the MDGs.

As the Global Burden of Disease Study 2013 found, mental and substance use disorders had increased over the preceding 3 years to account for 11% of DALYs (as compared with 7.8% in 2010) (GBD 2013 DALYs and HALE Collaborators, 2015), and these disorders alone accounted for 21.2% of YLDs worldwide in 2013 (Global Burden of Disease Study Collaborators, 2015). This makes mental illness a major challenge to any health system, and mental health a clear issue for development in low- and middle-income countries and in high-income countries (WHO World Mental Health Survey Consortium, 2004). Most recent research indicates that this impact may even be an underestimate (Vigo *et al.* 2016). Yet, less-developed countries in particular face a high treatment gap, meaning most people with a mental disorder do not receive any treatment at all and often face isolation, discrimination and violations of their human rights (Kohn *et al*. 2004).

In itself mental health is a prerequisite for physical health, and is strongly interlinked with other development factors such as poverty, work and economic growth or peace and justice. Mental health plays a key role in efforts to achieve social inclusion and equity, universal health coverage, access to justice and human rights, and sustainable economic development (World Health Organization, 2011). For example, poverty (goal 1) and mental illness are strongly linked, just as economic growth (goal 8) and safe and resilient cities and settlements (goal 11) depend on an overall mentally healthy society. As a cross-cutting issue mental health has relevance across the whole range of development (Chatterjee *et al*. 2014).

In mid-2014 the draft of the new generation of development goals was released by the UN Open Working Group. Compared with the MDGs, the SDGs now represented a broader range of development issues, aiming to maximise inclusion and to ‘leave no one behind’ (Open Working Group TUN, 2014). The draft set of 17 goals had been developed with little consultation of public and it included only one minor reference to mental health; civil society organisations, NGOs and academia had been largely ignored by the UN in this process. In this form the new development goals would have once more essentially ignored over 400 million people worldwide currently experiencing mental health issues (World Health Organization, 2013).

In this context a consortium was developed in 2014, led by King's College London and Maudsley International, recognising the need to challenge the post-2015 development agenda. A global initiative to strengthen mental health in the SDGs was created, called FundaMentalSDG, gathering leaders of global mental health from academia, civil society, and service user, carer and delivery organisations (www.fundamentalsdg.org). The FundaMentalSDG Steering Group advocated that UN member states should include two mental health targets and two indicators in the draft SDG health goal. The targets and indicators proposed were fully aligned with the WHO Global Mental Health Action Plan 2013–2020 (World Health Organization, 2013), and with the proposal presented by the UN Sustainable Development Solutions Network (UN SDSN) (Votruba *et al*. 2014).

## The contribution of FundaMentalSDG

With a series of co-ordinated global actions in 2014–2015, members of the FundaMentalSDG Steering Group approached international leaders and national mental health policy makers, in partnership with a growing civil society member network. The actions taken by the group included mailing of advocacy letters to all representatives of the UN member states (where contact details could be identified), active direct approaches of, and meetings with, individual key policy makers in the SDG process, advocacy presentations at national and international meetings, and others. International celebrities and members of the media picked up and supported this work, and in a few months the initiative gained global recognition (Luhrmann, 2015). In late 2014 former UN Secretary-General Kofi Annan stressed the significance of human rights violations related to people with mental disorders and pointed out the importance of mental health for global development (Kingsland, 2014). In his synthesis report on the post-2015 agenda in December 2014 UN Secretary-General Ban Ki-Moon included the need for the new development agenda to address universal health care coverage, access and affordability to reduce the burden of non-communicable diseases, including mental illness (United Nations, 2014).

Almost 1 year after FundaMentalSDG started its global effort, the UN member states’ negotiations on the new development agenda drew to a close. Despite all the joint efforts to strengthen mental health, that SDG draft did not give much room for hope. Competing with many other issues for global development, the mental health content of the draft SDGs had not been strengthened. Nevertheless, during the summer 2015 FundaMentalSDG once more reinforced its efforts and advocated at the national governments, highlighting the importance of mental health for global development to set the scene for the final negotiations at the UN in autumn 2015.

Then, in 2015 at its 70th session, the UN General Assembly adopted the resolution on the new 2030 Agenda for sustainable development. The final agenda ambitiously declares the aim to transform the world from 2015 to 2030 and defines global development in 17 goals and 169 targets. In a ground-breaking stride and much to the surprise of the global mental health community, the UN has not only included mental health in the agenda for the first time, but has moreover declared mental health a priority for global development (United Nations, 2015):

In the initial *declaration* part of the new SDGs (the preamble) the UN places mental health on an equal footing to physical health and calls upon its member states for equitable and universal access to health care and assurance of mental and social well-being, as well as promotion of mental health and well-being, and universal health coverage and access to quality health care:

‘*In these Goals and targets, we are setting out a supremely ambitious and transformational vision. We envisage a world (…) with equitable and universal access to quality education at all levels, to health care and social protection, where physical, mental and social well-being are assured’ (paragraph 7) (United Nations*, *2015**)*.

The UN further recognises mental illness as a *major challenge for sustainable development* and expresses its commitment to the prevention and treatment of non-communicable diseases, including behavioural, developmental and neurological disorders: ‘*To promote physical and mental health and well-being, and to extend life expectancy for all, we must achieve universal health coverage and access to quality health care. (…) We are committed to the prevention and treatment of NCDs, including behavioural, developmental and neurological disorders, which constitute a major challenge for sustainable development’ (paragraph 26) (United Nations*, *2015**)*.

Within goal 3 (the ‘*health goal’*), mental health is referred to three times, directly in the target to *‘reduce by one third premature mortality from non-communicable diseases through prevention and treatment and promote mental health and well-being’* (target 3.4); in the further target to *‘strengthen the prevention and treatment of substance abuse, including narcotic drug abuse and harmful use of alcohol’* (target 3.5); and is also implicitly included in universal health coverage to ‘*achieve universal health coverage, including financial risk protection, access to quality essential health-care services and access to safe, effective, quality and affordable essential medicines and vaccines for all’* (target 3.8) (United Nations, 2015). With this development agenda the UN has followed the recommendations of WHO and has made the *Global Goals* a clear statement for mental health in global development. This is an historic step for mental health, for which leaders in global mental health and non-governmental organisations like FundaMentalSDG have advocated over the past years.

## The impact and challenges of mental health advocacy work in the SDGs

FundaMentalSDG managed, for the first time, to globally unite a large number of mental health stakeholders with diverse positions and aims. In very short time, this advocacy initiative was able to mobilise for a clear aim with simple and clear policy demands: very specific SDG targets and indicators on suicide mortality and treatment coverage for people with severe mental illness.

An important question is: what was the impact of FundaMentalSDG in achieving the inclusion of mental health in the SDGs? The brief answer to that is that it is impossible to tease out the effect of one among several concurrent influences upon this process. Even more than national policy making, global development policy is subject to intense and long negotiations not only between different politics and positions, but also between a large number of stakeholders, and blocs of national and political interest groups. In the UN both nations and subject-oriented advocacy groups work hard to make their positions heard. Particularly for non-UN affiliates, the UN structures, committees, and policy making processes are rather opaque, and leave the mechanisms of political advocacy unclear. With many different organisations and individuals aiming for influence on the health goals, relative contribution through a non-profit organisation upon mental health policy making processes are hard to measure (Starling, 2010).

A few countries, mainly low- or middle-income countries, set a high priority on the SDGs and their outcomes, and some of these countries were supportive of the issue of mental health. In a few cases countries’ governments and/or UN SDG country representatives expressed explicit support for mental health, and are confirmed to have taken the issue further in the (un-)official UN negotiations. However, it is not entirely clear how the inclusion of mental health overall, and the particular wording of the targets, eventually came about.

In its first advocacy wave FundaMentalSDG advocated for the inclusion of one specific target within the health goal: ‘*The provision of mental and physical health and social care services for people with mental disorders, in parity with resources for services addressing physical health*.’ As well as the inclusion of two key indicators in line with the WHO Mental Health Action Plan 2013–2020:

‘*To ensure that service coverage for people with severe mental disorders in each country will have increased to at least 20% by 2020 (including a community orientated package of interventions for people with psychosis; bipolar affective disorder; or moderate-severe depression).*’ And ‘*To increase the amount invested in mental health to at least 5% of the total health budget by 2020, and to at least 10% by 2030 in each low and middle income country*’ (Thornicroft & Patel, 2014).

During consultations with policy makers in the course of the SDG negotiations, it became clear that less specific, open targets were more likely to be achieved within the draft set of targets. So FundaMentalSDG adapted its call and in a second wave advocated for a number of edits: (1) to include mental health in health goal 3; (2) to include full accordance with the WHO Global Action Plan for the Prevention and Control of Non-Communicable Diseases and the WHO Mental Health Action Plan 2013–2020; and (3) to include universal health coverage for mental disorders (Votruba *et al*. 2015).

The eventual inclusion of mental health in the SDGs can be seen as a process with two layers. On one level, a range of international organisations have been advocating directly and indirectly for the overall inclusion of mental health in the SDGs, such as the World Psychiatric Association (WPA), or such as the Christoffel Blindenmission (CBM) which has focus on disability. Many organisations working for the promotion of overall health, women, children, victims of war crimes, etc. also support mental health inclusion in their wider focus. A second level comprises well-placed and established organisations within the inner (rather opaque) circle of UN diplomacy, such as WHO, and well-connected individuals. It is likely that their sustained positions, and long years of advocacy work have also played a key role in the process. In addition, within the SDG stakeholders’ positions there is an emerging view in relation to mental disorders as forms of disability, and the well-organised disability advocacy community is now beginning to take a more positive and inclusive approach to psychosocial disabilities.

In terms of measuring the impact of FundaMentalSDG, comparing the situation of mental health in the SDGs before and after the efforts of FundaMentalSDG indicates the likelihood of substantial achievement. When FundaMentalSDG began its efforts in summer 2014 the draft 2030 Development Agenda did not make substantial reference to mental health. Within 1 year of intense advocacy work by this global mental health initiative the UN SDG draft changed materially and mental health was included and highlighted multiple times, both in the preamble and in health Goal 3. This indicates that it is reasonable to consider that together with the efforts of WHO, FundaMentalSDG played a significant role in the inclusion of mental health in the UN SDGs, and particularly with regards to the inclusion of the specific mental health targets.

## Learnings from the FundaMentalSDG initiative

The work of the global initiative FundaMentalSDG has provided many insights into the UN system and processes, and lessons for mental health advocacy work on the global and national level. We would like to share learning on: (1) what the UN needs to change to make the SDG process more equitable for NGOs; and (2) what the global mental health community can do to make mental health advocacy work more efficient.

(1) What can the UN do to support a fairer and more equitable advocacy work in the UN SDGs, which is more representative of civil society and thus overall contributes to more sustainability?

A first big challenge lies in the setup of the SDG process and system. The SDGs are designed as a set of goals for nations, to be decided by nations, without any major input from civil society or academia. This in itself is a problem for NGOs who want to represent their issues in the process. But it is also reflected in limited public communication on opportunities to influence the SDGs on the side of the UN and its member states, which acts as another barrier. In many UN countries, the SDGs are not promoted as a policy issue and public communication from the UN and its member states is lacking when it comes to details of the process. This contributes to the third challenge, a lack of awareness of the SDG's relevance and possible inputs on the side of civil society/academia/NGOs, etc. NGOs of all areas of advocacy have experienced these three limitations throughout the SDG process.

To make the SDG's goals truly for the people, and to ‘*leave no one behind’*, the UN needs to ensure that populations are not only represented through their governments’ representatives, but also adequately through representatives of civil society. NGOs and representatives should be actively invited by national governments and the UN to contribute to inputs, in a larger, more direct and more frequent manner than has been the case for the 2015 SDG process. A core prerequisite for this, and for more effective civil society involvement in the SDG process in general, is that the overall low priority of the SDGs is increased by and within the UN member states, for both SDG-funding countries and low and middle-income countries.

(2) What can NGOs do to contribute to a more efficient and effective advocacy work in mental health? The work of FundaMentalSDG shows the value of the mobilisation and unification in mental health.

For a strong and efficient impetus for global mental health, the global mental health community should consider the following lessons: (a) Stakeholders need to collaborate on and consolidate a unified voice; (b) They need to raise clear and simple policy demands for specific mental disorders, services or system changes; (c) They need to work on establishing continuous and trusting relationships with policy makers to bring mental health into the global policy agenda. And within this route (d) Civil society, NGOs and academia need to take their share of responsibility to promote the importance of the SDGs, and the need for influence, towards their citizens and member states.

## The crucial next step: strong mental health indicators

With mental health being included in goals and targets of the new Development Agenda, what are the next steps for the UN? As the UN Secretary General has pointed out in his 2014 Synthesis report, rigorous and robust measurement will determine the success of the Global Goals. All envisaged improvements in global development need to be measured and tracked by reliable systematic data and indicators, which include a level of disaggregation to address inequities and inequalities.

Based on the draft of the UN Inter-Agency Experts Committee on the indicators (IAEG-SDG) the UN is developing indicators, with a final decision expected later in 2016. For mental health it is of particular importance that the indicators make a clear reference to mental disorders, to avoid the risk of being disregarded compared with targets which do have clear indicators.

WHO has proposed two indicators to strengthen mental health in the SDGs, which are fully aligned with the WHO Global Mental Health Action plan, both within the health goal: (1) suicide rate; and (2) service coverage (proportion treated) of persons with severe mental illness (World Health Organization, 2015). FundaMentalSDG fully supports these two indicators, especially as no additional effort is required from the UN/WHO countries to comply with the indicators since they have already agreed to the data collection for these indicators within the WHO Mental Health Action plan. (World Health Organization, 2013).

The current list of indicators negotiated by the IAEG for adoption by the UN General Assembly includes an indicator on suicide mortality rate (indicator 3.4.1). For target 3.8 universal health coverage, the IAEG proposed an indicator for service coverage (indicator 3.8.1). FundaMentalSDG supports the WHO's suggestion to explicitly include treatment for people with severe mental disorders (SMD) under that indicator.

Without clear and detailed indicators, mental health lacks metrics to measure progress. In order to better reflect inequalities and include people with mental illness, the UN Sustainable Development Solutions Network (UN SDSN) has laid out the need for the UN to identify levels of disaggregation of certain relevant indicators (UN SDSN, 2015). FundaMentalSDG supports the disaggregation of the indicators to ensure measurable progress, and calls for the support of the UN to give relevance to people with mental disorders and psychosocial disabilities.

## Conclusions

Mental health has come a long way from its exclusion from the MDGs to the 2015 Development Agenda. In the new SDGs, the UN has finally defined mental health as a global development priority, and set the scene for an ambitious plan to tackle the world's challenges in the coming 15 years.

Now NGOs, civil society organisations and academia must remind the UN that action needs to follow formal commitment. The Global Goals require a detailed action plan, and the implementation of the health targets needs to be defined and measured by indicators. Mental health in particular needs specific, clear indicators to identify, measure and implement mechanisms for the strengthening and scale-up of mental health systems. This applies particularly for low-income countries where coverage and provision of mental health services are widely lacking. Mental health is a challenge for global development not only in itself, but also influences many other development targets as a cross-cutting issue.

The UN, as well as NGOs, civil society organisations and academia, need to recognise the relevance of mental health to many of the SDGs, and must strengthen mental health with clear indicators. In addition, the UN needs to disaggregate the indicators to ensure equity in health. In the coming 14 years, the UN and its member states, NGOs and academia need to measure and monitor the data from the indicators to ensure that the SDG targets are met.

NGOs and academia need to maintain momentum and, as guardians of the SDGs, hold the UN to account. Lastly, the UN member states need to commit to their 2030 agenda promise to *leave no one behind* and to ensure prevention and treatment, and inclusion within universal health coverage for people with mental disorders and psychosocial disabilities.
